# Release and extraction of retained subfoveal perfluorocarbon liquid facilitated by subretinal BSS, vibration, and gravity: a case report

**DOI:** 10.1186/s12886-020-01698-1

**Published:** 2020-10-23

**Authors:** Kosuke Takahashi, Shuhei Kimura, Mio Morizane Hosokawa, Yusuke Shiode, Shinichiro Doi, Ryo Matoba, Yuki Kanzaki, Yoshihiro Yonekawa, Yuki Morizane

**Affiliations:** 1 Department of Ophthalmology, Okayama University Graduate School of Medicine, Dentistry and Pharmaceutical Sciences, 2-5-1 Shikata-cho Kita-ku, Okayama City, Okayama, 700-8558 Japan; 2 Wills Eye Hospital, Mid Atlantic Retina, Thomas Jefferson University, Philadelphia, PA USA

**Keywords:** Case report, Perfluorocarbon, Retinal detachment, Subretinal injection, Vitreoretinal surgery

## Abstract

**Background:**

Perfluorocarbon liquid (PFCL) is an effective surgical adjuvant in performing vitrectomy for severe vitreoretinal pathologies such as proliferative vitreoretinopathy and giant retinal tears. However, subretinal retention of PFCL can occur postoperatively and retained PFCL causes severe visual disorders, particularly when PFCL was retained under the fovea. Although several procedures have been proposed for subfoveal PFCL removal, such as direct aspiration or submacular injection of balanced salt solution (BSS) to dislodge the subfoveal PFCL, the retinal damage associated with these procedures has been a major problem. Here, we report a case of subfoveal retention of PFCL for which we performed a novel surgical technique that attempts to minimize retinal damage.

**Case presentation:**

A 69-year-old man presented with subfoveal retained PFCL after surgery for retinal detachment. To remove the retained PFCL, the internal limiting membrane overlying the subretinal injection site is first peeled to allow low-pressure (8 psi) transretinal BSS infusion, using a 41-gauge cannula, to slowly detach the macula. A small drainage retinotomy is created with the diathermy tip at the inferior position of the macular bleb, sized to be slightly wider than that of the PFCL droplet. The head of the bed is then raised, and the surgeon gently vibrates the patient’s head to release the PFCL droplet to allow it to migrate inferiorly towards the drainage retinotomy. The bed is returned to the horizontal position, and the PFCL, now on the retinal surface, can be aspirated. The subfoveal PFCL is removed while minimizing iatrogenic foveal and macular damage. One month after PFCL removal, the foveal structure showed partial recovery on optical coherence tomography, and BCVA improved to 20/40.

**Conclusion:**

Creating a macular bleb with low infusion pressure and using vibrational forces and gravity to migrate the PFCL towards a retinotomy can be considered as a relatively atraumatic technique to remove subfoveal retained PFCL.

**Supplementary information:**

**Supplementary information** accompanies this paper at 10.1186/s12886-020-01698-1.

## Background

Perfluorocarbon liquid (PFCL) is an effective surgical adjuvant in performing vitrectomy for severe vitreoretinal pathologies such as proliferative vitreoretinopathy and giant retinal tears [1, 2]. However, subretinal retention of PFCL can occur in 1–11% of cases [3, 4]. There may be toxic effects on the retinal pigment epithelium (RPE) and photoreceptors, [5] and retained PFCL can be visually significant, particularly when loculated under the fovea [6].

Several procedures have been proposed for subfoveal PFCL removal, including direct aspiration by using cannulas to puncture through the fovea [7]. This may lead to scotomas and complications such as macular hole, submacular hemorrhage, subfoveal choroidal neovascularization, and submacular fibrosis due to photoreceptor and RPE cell damage [7, 8]. Another reported technique utilizes submacular injection of balanced salt solution (BSS) to dislodge the subfoveal PFCL, with or without extraction of the PFCL [9, 10]. This is likely less traumatic compared to the former technique, but forceful submacular BSS injection can create macular holes and photoreceptor damage if the injection pressure is high [10–13]. Displacement of the subfoveal PFCL to the extrafoveal area without aspiration of the PFCL requires postoperative postural positioning, and the displaced PFCL may migrate back to the fovea [6].

An atraumatic procedure for subfoveal PFCL removal without the above limitations would therefore be beneficial. Herein, we describe a novel surgical technique for subfoveal PFCL removal that attempts to minimize photoreceptor and RPE damage.

## Case presentation

### Presentation, history, and the initial surgery for retinal detachment

**Fig. 1:**
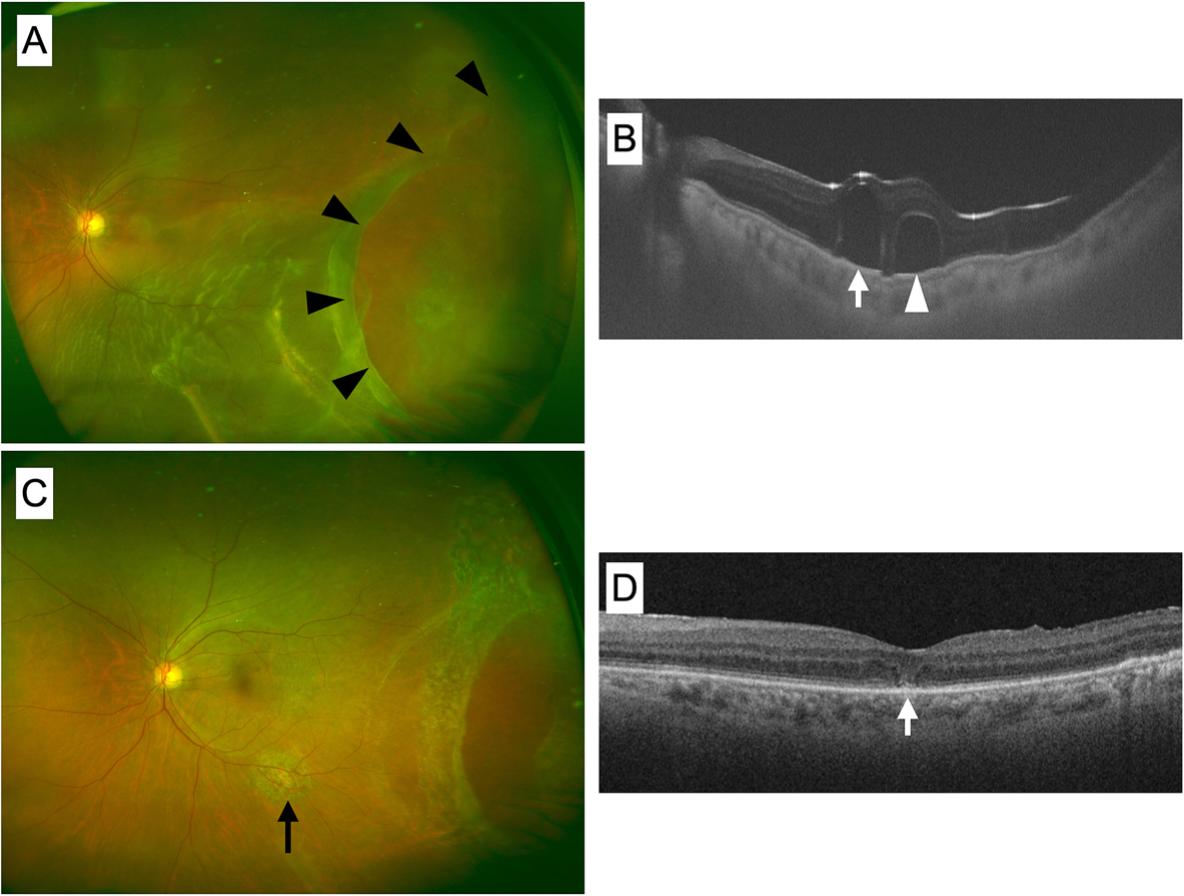
Preoperative and postoperative color fundus images and swept source optical coherence tomography. **a** A preoperative color fundus image shows a retinal detachment with a temporal giant retinal tear (arrowheads). **b** Swept-source optical coherence tomography (OCT) B-scan images obtained under intraocular gas on postoperative day 1 shows subretinal retention of two perfluorocarbon liquid (PFCL) droplets. The droplet indicated by the arrow is located under the fovea; that indicated by the arrowhead is located in the parafoveal area. **c** Color fundus image obtained 1 month after PFCL removal. The retina is reattached. The arrow points to the drainage retinotomy created during the first surgery, which was used to extract the subretinal PFCL droplets to the retinal surface. **d** OCT B-scan image obtained 1 month after PFCL removal shows resolution of the subretinal PFCL. The outer retinal microstructure of the fovea is not completely restored, but visual acuity was 20/40 (arrow)

A 69-year-old man presented with a temporal giant retinal tear in the left eye with a macula-involving retinal detachment. His best-corrected visual acuity (BCVA) was 20/160 (Fig. 1a). We performed 25-gauge pars plana vitrectomy with combined phacoemulsification. PFCL was used to flatten the retina, but there was residual subretinal fluid after PFCL removal, so a small drainage retinotomy was created outside the arcade vessel at 6 o’clock to drain the remaining fluid, and photocoagulation was performed, and 20% sulfur hexafluoride (SF_6_) was used for tamponade.

### The second surgery to remove subretinal PFCL droplets

On the first postoperative day, swept-source optical coherence tomography (OCT) obtained through the gas bubble showed subretinal PFCL droplets, one of which was retained under the fovea (Fig. 1b). We performed a second surgery to remove the retained PFCL 5 days later. Four subretinal PFCL droplets were identified intraoperatively. Of these, two extrafoveal droplets were removed by direct aspiration with a 41-gauge cannula. For removal of the subfoveal and parafoveal PFCL, we created a macular detachment via subretinal BSS injection at 8 psi, such that the area of the detachment included both the PFCL droplets and the previous retinotomy, which was conveniently created at the inferior position during the first surgery. The head of the bed was elevated, and the patient’s head was carefully vibrated for 10 s. The subfoveal PFCL released onto the retinal surface and was removed. Subsequently, we confirmed that the second, parafoveal, droplet was still subretinally retained by applying PFCL on the retinal surface to better delineate its spherical shape. The same maneuver was repeated, which released the second droplet. Fluid–air exchange, retinopexy, and air–20% SF_6_ gas exchange were performed (see Additional file 1).


**Additional file 1** Surgical video of subfoveal perfluorocarbon liquid removal using subretinal balanced salt solution, vibrational forces, and gravity.

### Post-operative recovery of the macula

**Fig. 2:**
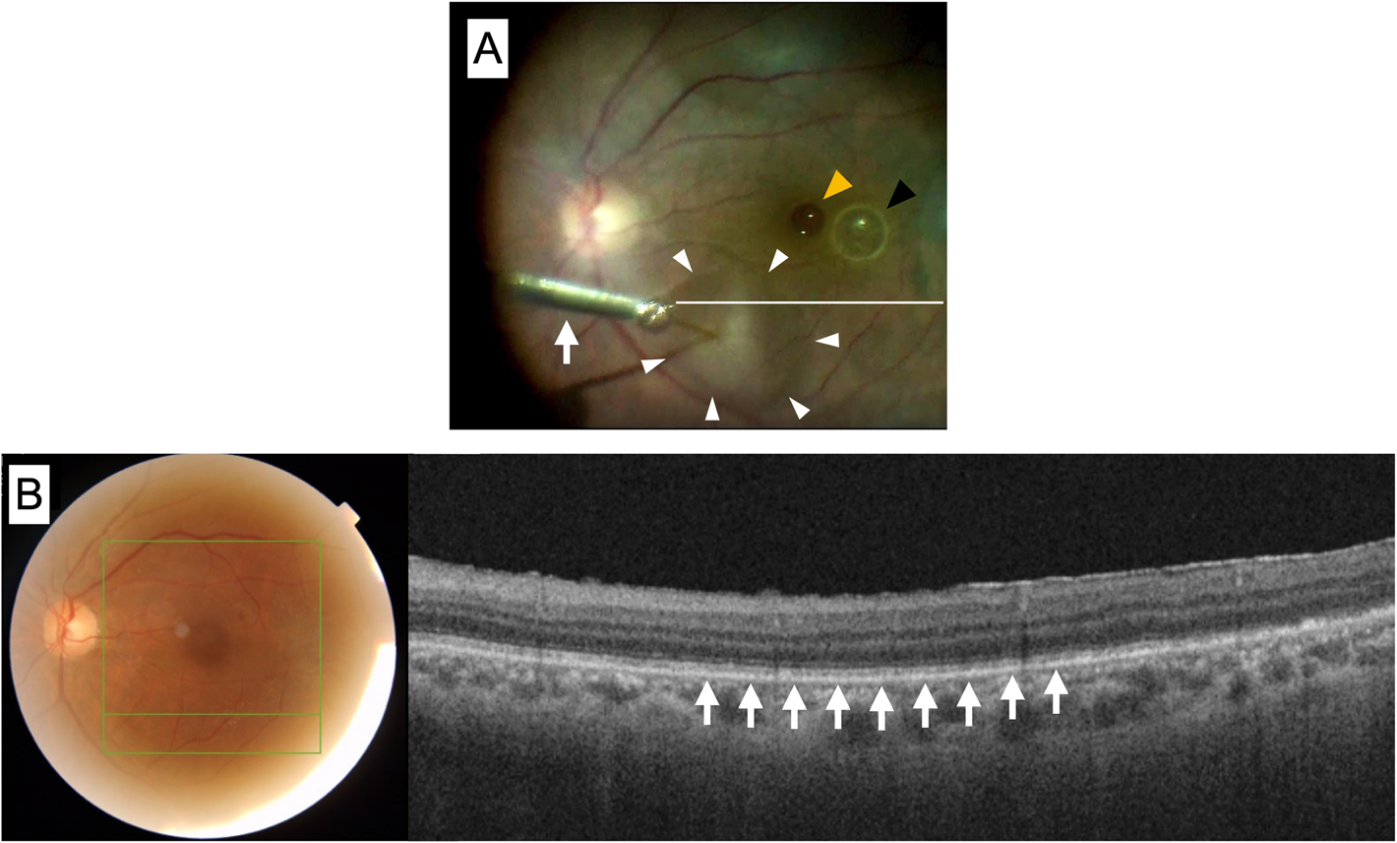
Subretinal injection of balanced salt solution (BSS) and postoperative optical coherence tomography (OCT). **a** An intraoperative image demonstrating the initiation of subretinal infusion of BSS using a 41G cannula (arrow) (Additional file 1). The arrowheads indicate the border of the bleb. The white line corresponds to the OCT scan in (**b**). The yellow and black arrowheads indicate the subfoveal and parafoveal PFCL droplets, respectively. **b** OCT image scanned at the horizontal line in Fig. **a**, 1 month after PFCL removal. The outer retinal structure is continuous (arrows)

One month after PFCL removal, the foveal structure showed partial recovery on OCT, and BCVA improved to 20/40 (Fig. 1d). OCT of the area of the macular bleb showed no anatomic damage to the photoreceptors and RPE (Fig. 2).

### Detailed explanation of surgical technique for subfoveal PFCL removal

**Fig. 3:**
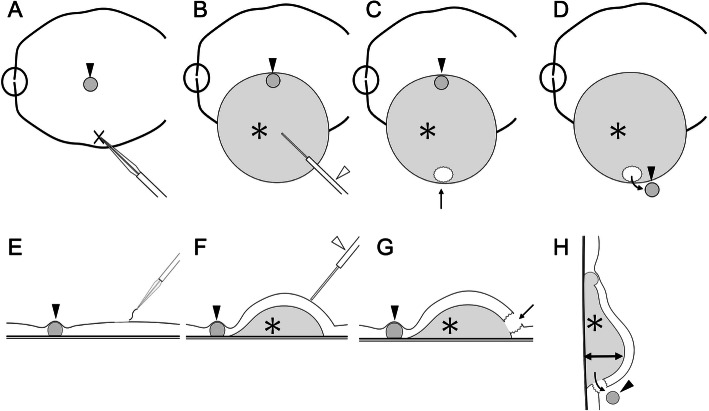
Schematic of a novel surgical technique for subfoveal perfluorocarbon liquid (PFCL) removal. **e, f, g**, and **h** show vertical cross-sections of **a, b, c**, and **d**, respectively. The black arrowheads indicate the local of the PFCL droplet. **a** and **e** The internal limiting membrane at the site for subretinal injection of BSS (cross mark). **b** and **f** Subretinal bleb (asterisk) is induced by subretinal BSS injection using a 41-gauge cannula (white arrowhead) at a pressure of 8 psi. **c** and **g** An small drainage retinotomy (arrow) is created at the inferior end of the bleb (asterisk). **d** and **h** The patient’s position is changed from supine to upright. When the patient is placed in the upright position, the subretinally injected BSS gathers inferiorly from gravity, while the height of the retinal detachment below the PFCL droplet increases (double headed arrow). Then, the head is vibrated by the surgeon to facilitate migration of the subfoveal PFCL to the retinal surface through the retinotomy.


The first step is to create a submacular bleb with BSS. In order to minimize the injection pressure of the BSS to avoid diffuse photoreceptor and RPE damage, the internal limiting membrane (ILM) at the subretinal injection site can be focally peeled before placement of the cannula (Fig. 3a, e) [11–13]. BSS is inserted into the Viscous Fluid Control system (VFC; Alcon Laboratories, Fort Worth, TX), which is connected to a 41-gauge cannula (MedOne, Sarasota, FL). The cannula is placed at the ILM opening, and foot pedal control is used to transfuse the BSS at a low injection pressure of 8 psi through the retina (without having to penetrate through), to create a submacular BSS bleb (Fig. 3b, f). By pre-peeling the ILM, the BSS can permeate through the neurosensory retina into the subretinal space, even without puncturing through with the cannula tip. The macular detachment should extend across the posterior pole beyond the arcade vessels, especially inferiorly. Subsequently, a small drainage retinotomy is created with a diameter slightly larger than that of the PFCL droplet at the inferior edge of the bleb, outside of the arcades (Fig. 3c, g). The infusion line is then clamped, and removed from the self-sealing cannula. All cannulas are inspected to be water-tight. The head of the patient’s bed is then raised from supine to upright position, while ensuring that vitrectomy unit cords and anesthesia-related apparatuses are not compromised. With the head now elevated, the height of the macular detachment inferior to the PFCL increases due to gravitational forces (Fig. 3h). The surgeon then carefully vibrates the patient’s head manually for approximately 10 s to release the PFCL from the subfoveal position, which then finds its way out of the inferior drainage retinotomy (Fig. 3d, h). The patient is returned to the supine position, and the PFCL droplet on the retinal surface is aspirated. This is followed by fluid–air exchange and photocoagulation around the drainage retinotomy. At the end of the surgery, air is replaced with 20% SF_6_ gas (see Additional file 2).

**Additional file 2** Animation of a novel surgical technique for subfoveal perfluorocarbon liquid removal using subretinal balanced salt solution, vibrational forces, and gravity.

## Discussion and conclusions

We describe a new surgical technique to remove subfoveally retained PFCL, with the goal of minimizing theoretical iatrogenic effects to the foveal and macular outer retina. Several observations were made during this procedure.

**Fig. 4:**
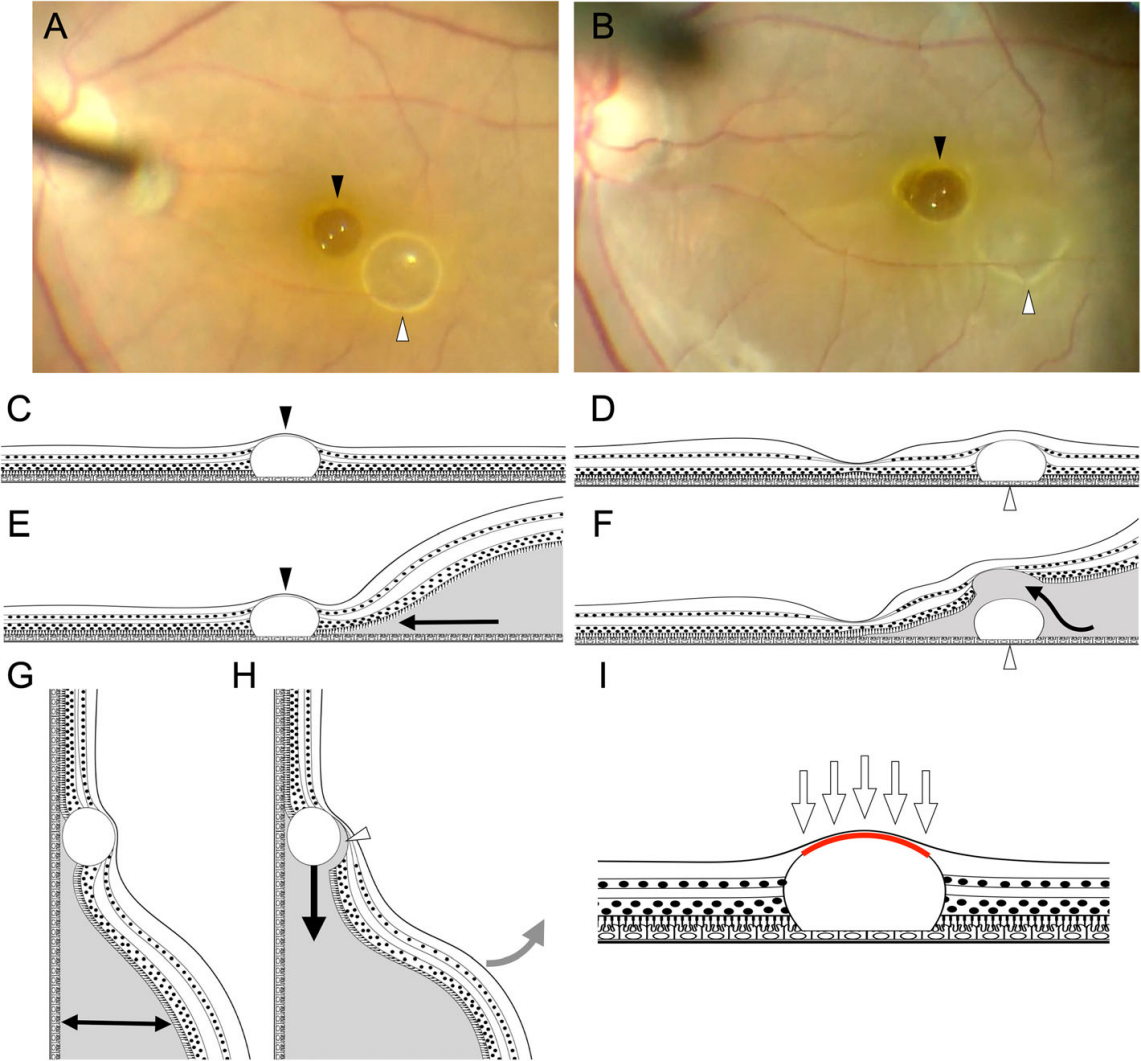
Subfoveal perfluorocarbon (PFCL) liquid is adherent to the fovea during macular bleb creation. **a** Intraoperative image prior to creation of the macular bleb. The black and white arrowheads indicate the subfoveal and parafoveal PFCL droplets, respectively. **b** Intraoperative image after the creation of the macular bleb. The area of macular detachment includes both PFCL droplets. The retina was easily lifted off the parafoveal PFCL (white arrowhead) with the bleb creation, and the outline of the parafoveal PFCL droplet is now more obscured compared to Fig. **a**. However, the retina does not lift off the subfoveal PFCL (black arrowhead). The outline of the subfoveal droplet remains well delineated. **c** Schematic of subfoveal PFCL (black arrowhead), showing possible contact with the internal limiting membrane (ILM) via compressed or separate Müller cell cones. **d** On the other hand, the parafoveal PFCL (white arrowhead) appears to stay well beneath the outer retina and may make contact with the inner retinal layer, but not the ILM. **e** The arrows indicate the flow of the injected BSS. At the fovea, BSS does not significantly flow between the PFCL (black arrowhead) and the retina due to adhesion between the two. **f** However, at the parafoveal area, BSS flows easily between the PFCL (white arrowhead) and the retina, as shown by the arrow (**f**). **g** When the patient is repositioned vertically, the subretinally injected BSS gathers inferiorly, while the height of the retinal detachment inferior to the PFCL increases (double headed arrow). **h** Vibrational forces (gray arrow) break the adhesion between the PFCL and the retina (arrowhead). This causes the PFCL droplet to migrate inferiorly (arrow). **i** Adhesion between the surface of the subfoveal PFCL and Müller cell cones/ILM. To break this adhesion, a force greater than the intraocular pressure (arrows) multiplied by the area of the adhesion (red line) is required

In order to displace the PFCL droplets, we created a macular detachment with BSS and encountered two phenomena. First, although the macular detachment was easily created over the PFCL droplet in the parafoveal area, it could not be extended above the subfoveal droplet (Fig. 4a, b; Additional file 1). A possible reason is that the retinal tissue in contact with the PFCL droplets differed between the subfoveal and parafoveal droplets. As shown in Figs. 1 and 4, the parafoveal PFCL stays mostly within the outer retina, whereas the subfoveal PFCL is draped by very thin tissue [14]. The inner retinal layers are absent at the fovea, except Müller cell cones. Therefore, we speculate that the interface between the PFCL and thinner foveal tissue may be stronger than the extrafoveal photoreceptors. This adhesive force, whether it may be mechanical from the draping by the thin foveal tissue, and/or a vacuum-like state from surface tension, prevented BSS from flowing above the subfoveal PFCL. On the other hand, there were no significant adhesive forces between the extrafoveal PFCL and outer retina, which permitted easy separation. To create macular detachments over subfoveal PFCL, the subretinal injection is required to be performed at a pressure so that the force of injected BSS flow becomes greater than the intraocular pressure multiplied by the area of adhesion between the subfoveal PFCL and fovea (Fig. 4i). The intraocular pressure can therefore be lowered to facilitate this step.

Second, the injection pressure (8 psi) alone failed in displacing both the subfoveal and parafoveal PFCL droplets. To release the droplets from the RPE, we utilized gravity and vibration to mobilize the subretinal PFCL (Fig. 4g, h; Additional files 1 and 2). To utilize gravity, just as the rotation of surgical table during vitrectomy for giant retinal tear, [15, 16] we thought that it was effective to change the patient’s position from supine to upright by moving the head of the patient’s bed. When moving the head of the patient’s bed, care should be taken to ensure that infusion and anesthesia lines are secure and the patient should be strapped securely depending on the method of anesthesia and the patient’s physical attributes. The vibration should be gentle enough not to cause iatrogenic complications, and not recommended in young children, elderly patients, or anyone with intracranial or cervical susceptibilities, and intubation would be a contraindication. Patients undergoing vitreoretinal surgery in Japan often receive local anesthesia without any systemic anesthetics, and are fully alert. This may not be the practice pattern in many settings, in which case raising the head of the bed may not be safe. Furthermore, not all surgical beds have the ability to raise the patient’s head upright. In such settings, we recommend temporal drainage retinotomies, and tilting the head temporally to use the same principles. The bleb should be large enough, and retinotomy far temporal enough, to assure that a scotoma does not develop. Tilting the chin up and head reclined further with a superior retinotomy may be an option also. Whichever procedure is chosen, the number of head tilts and shakes should be minimized to avoid discomfort to the patient.

Creation of the submacular bleb is a key component of this procedure. Prior peeling of the ILM (which accounts for half of retinal stiffness [17]) at the subretinal injection site allows transretinal infusion of BSS into the subretinal space using a low injection pressure (6–8 psi), even without the cannula puncturing through the retina [11, 12]. We confirmed in non-human primate eyes that 8 psi prevents diffuse photoreceptor and RPE damage that may accompany subretinal injections at higher infusion pressures [13]. Consistent with these data, we were likely able to minimize cellular damage to the macula (Fig. 2b).

In conclusion, we present a novel surgical technique utilizing focal ILM peeling, subretinal BSS, gravity, and vibrational forces to release and extract subfoveally retained PFCL. Since the retained subfoveal PFCL may have toxic effects on the RPE and photoreceptors, [5] the PFCL should be removed as soon as its existence is identified. Future studies would be warranted to generate more efficacy and safety data, as well as to expand our understanding of manipulations in the submacular space with modern instrumentation and surgical techniques.

## Patient perspective

The patient was content with the improvement of vision in the left eye, which has allowed him to continue his daily activities.

## Data Availability

Data sharing is not applicable to this article as no datasets were generated or analysed during the current study.

## Abbreviations

BCVA: Best corrected visual acuity
BSS: Balanced salt solution
ILM: Internal limiting membrane
OCT: Optical coherence tomography
PFCL: Perfluorocarbon liquid
RPE: Retinal pigment epithelium
SF_6_: Sulfur hexafluoride
VFC: Viscous Fluid Control system
